# Three-Dimensional Geometric Analysis of Balloon-Expandable Covered Stents Improves Classification of Complications after Fenestrated Endovascular Aneurysm Repair

**DOI:** 10.3390/jcm11195716

**Published:** 2022-09-27

**Authors:** Claire van der Riet, Richte C. L. Schuurmann, Eric L. G. Verhoeven, Athanasios Katsargyris, Ignace F. J. Tielliu, Timothy Resch, Reinoud P. H. Bokkers, Jean-Paul P. M. de Vries

**Affiliations:** 1 Department of Surgery, Division of Vascular Surgery, University Medical Center Groningen, 9700 RB Groningen, The Netherlands; 2 Multimodality Medical Imaging Group, Technical Medical Centre, University of Twente, 7522 NB Enschede, The Netherlands; 3 Department of Vascular and Endovascular Surgery, General Hospital Nuremberg, Paracelsus Medical University, 90471 Nuremberg, Germany; 4 Department of Vascular Surgery, Rigshospitalet, Faculty of Health and Medical Sciences, University of Copenhagen, 2200 Copenhagen, Denmark; 5 Department of Radiology, Medical Imaging Center, University Medical Center Groningen, University of Groningen, 9713 GZ Groningen, The Netherlands

**Keywords:** abdominal aortic aneurysm, fenestrated endovascular aneurysm repair, balloon-expandable covered stent, geometry, 3D imaging, 3D reconstructions

## Abstract

In balloon-expandable covered stent (BECS) associated complications after fenestrated endovascular aneurysm repair (FEVAR), geometric analysis may determine the cause of failure and influence reintervention strategies. This study retrospectively classifies BECS-associated complications based on computed tomographic angiography (CTA) applied geometric analysis. BECS-associated complications of FEVAR-patients treated in two large vascular centers between 2012 and 2021 were included. The post-FEVAR CTA scans of complicated Advanta V12 BECSs were analyzed geometrically and complications were classified according to its location in the BECS. BECS fractures were classified according to an existing classification system. In 279 FEVAR-patients, 34 out of the 683 included Advanta V12 BECS (5%) presented with a complication. Two Advanta V12 complications occurred during the FEVAR procedure and 32 occurred during follow-up of which five post-FEVAR CTA scans were missing or not suitable for analysis. In the remaining 27 BECSs complications were classified as (endoleaks (n = 8), stenoses (n = 4), occlusions (n = 6), fractures (n = 3), and a combination of complications (n = 6)). All BECSs associated complications after FEVAR with available follow up CTA scans could be classified. Geometric analysis of BECS failure post-FEVAR can help to plan the reintervention strategy.

## 1. Introduction

Fenestrated endovascular aneurysm repair (FEVAR) is a well-established treatment modality for patients with a juxtarenal abdominal aortic aneurysm (JAAA) [1,2]. The custom-made fenestrated stent graft (FSG) enables juxta- and suprarenal sealing while maintaining blood flow to the visceral arteries through the fenestrations. Balloon-expandable covered stents (BECS) used as bridging stents between the fenestrations and the target arteries are essential to avoid endoleaks and to secure patency of the visceral arteries but form a potential source of complications that needs attention during follow-up.

Reinterventions have been reported in 20% to 39% of FEVAR patients after 2.0 to 7.5 years of follow-up [3,4,5,6]. Approximately half of these reinterventions are performed for BECS-associated complications [3]. These complications can be divided into endoleak, stenosis and/or occlusion, or stent fracture with occurrence rates of 12%, 6%, and 6%, respectively [3,6]. Most durability studies, however, are limited in including heterogeneous groups of patients and BECSs, with short-term follow-up, and/or a mixture of fenestrated and branched endovascular repair [7].

In case of an endoleak at follow-up, it can be difficult to detect which fenestration (and subsequent BECS) causes the problem [8]. Moreover, not all BECS stenoses or occlusions are caused by the same mechanism. It is important to identify the exact origin of BECS failure as it influences the eventual reintervention strategy. Dedicated software for quantification and visualization of the three-dimensional (3D) geometry of a BECS may be supportive to classify the mode of BECS failure and to guide reintervention strategy [9,10]. This paper provides an overview of modes of BECS failure and classifies Advanta V12 (Atrium Medical Corporation, Merrimack, NH, USA) BECS with complicated follow-up according to these modes of failure.

## 2. Materials and Methods

### 2.1. Study Population

The dataset of this retrospective study comprised patients treated with FEVAR between January 2012 and April 2021 in the University Medical Center Groningen, the Netherlands, and between January 2012 and December 2015 in the General Hospital, Nuremberg, Germany. The FEVAR-patients treated between January 2012 and December 2015 have been described in a previous study [11]. All patients underwent primary FEVAR to treat a JAAA or to treat a previous failed EVAR and at least one fenestration had to be stented with an Advanta V12 BECS. Only the Advanta V12 BECSs were analyzed in the current study to keep the group as homogeneous as possible.

Patient data were processed in agreement with the Declaration of Helsinki’s ethical principles for medical research involving human subjects. Clinical data were retrospectively collected from the electronic patient records and were registered in a Research Electronic Data Capture (REDCap, version 8.10.18; Vanderbilt University, Nashville, TN, USA). All electronic patient records were searched for FEVAR procedural details such as type of FSG, number of fenestrations, fenestration dimensions (width × height), fenestrations stented with Advanta V12 BECS, Advanta V12 BECS size (diameter × length), and BECS-associated complications on the completion angiography. Post-FEVAR computed tomograpic angiography (CTA) scan and duplex ultrasound (DUS) were reviewed for Advanta V12 BECS-associated complications. The endpoint was one (or a combination) of the following Advanta V12 BECS-associated complications: endoleak, stenosis or occlusion, and fracture. For Advanta V12 BECS with a complicated follow-up, geometric analysis was performed on post-FEVAR CTA scan(s).

### 2.2. CTA Scan Protocol

In both vascular centers, images were acquired on a 384-slice CT scanner (Somatom, Siemens Healthineers, Erlangen, Germany). Scan parameters of the General Hospital Nuremberg CTA protocol were: 100 kV tube voltage with variable mAs up to a maximum of 600 mAs tube current time product, 0.8 mm pitch, and 230 mm detector collimation. Per CTA scan, 100 mL (4 mL/second) of diluted contrast (Xenetrix 300; Guerbet, Sulzbach, Germany) was administered intravenously and scanned in the arterial phase. Scan parameters of the University Medical Center Groningen CTA protocol were: 80 kV tube voltage with variable mAs up to a maximum of 850 mAs tube current time product, 0.8 mm pitch, and 230 mm detector collimation. Per CTA scan, 100 mL (4 mL/second) of diluted contrast (Iomeron 350; Imaging GmbH, Konstanz, Germany) was administered intravenously and scanned in the arterial phase. Images were reconstructed to 0.75 or 2.0 mm slice thickness using a medium-smooth convolution kernel.

### 2.3. Geometric Analysis of BECS

BECS geometry was analyzed with a combination of CTA-derived measurements and dedicated Flare Geometry Analysis (FGA) software (Endovascular Diagnostics BV, Bussum, The Netherlands) in order to define the mode of failure of the BECS-associated complications.

**Figure 1:**
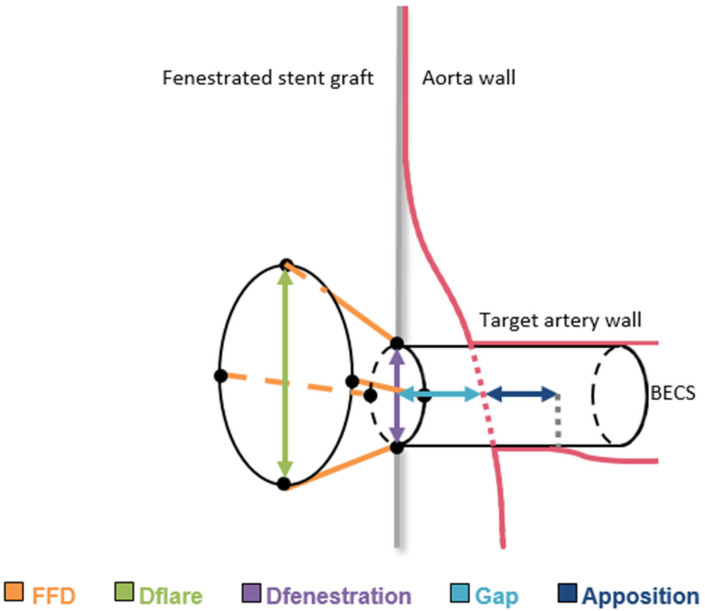
Schematic presentation of the fenestrated stent graft (FSG) in the aorta and the balloon-expandable covered stent (BECS) connecting the target artery to the FSG. The geometric parameters, flare-to-fenestration distance (FFD), diameter of the flare (Dflare), and diameter of the fenestration (Dfenestration), gap distance (gap), and circumferential apposition (apposition) quantify BECS geometry.

The first step in BECS analysis was performing post-FEVAR CTA scan measurements in a 3mensio 10.1 Vascular workstation (Pie Medical, Bilthoven, The Netherlands) following a predefined measurement protocol. The measurements were performed by an experienced reader (CR) and confirmed by a second experienced reader (RS). A centerline was constructed in the lumen of the FSG and separate centerlines were constructed of the Advanta V12 BECSs and target arteries. The centerlines were placed semi-automatically with manual correction of misplaced center lumen points. Thereafter, eight Cartesian coordinate markers were placed: four markers at the flared end of the BECS and four at the level of the fenestration. The following BECS parameters were measured on the snake view of the target artery in 3mensio (Figure 1):

1. Gap bridged by the BECS, defined as the centerline distance from the wall of the FSG to the orifice of the target artery at the aortic wall.
2. Apposition between the BECS and the target artery, defined as the centerline distance from circumferential apposition between the BECS and the target artery to the distal end of circumferential apposition of the BECS.

After CTA derived measurements, the centerlines and the coordinate markers were exported from 3mensio and imported into the FGA software for quantification and visualization of 3D BECS geometry. The FGA methodology has been described in detail and has been validated in-vitro in a previous publication [10]. The following BECS parameters were calculated with the FGA software (Figure 1):

1. Flare-to-fenestration distance (FFD), defined as the length of the BECS from the proximal end of the flare to its corresponding point at the fenestration. This is calculated for the entire circumference of the flare and reported as the shortest FFD.
2. Diameter of the BECS at the proximal end of the flare (Dflare), reported as the minimum and maximum diameters.
3. Diameter of the BECS at the fenestration (Dfenestration), reported as the minimum and maximum diameters.

In addition, BECS D-ratios at the proximal end of the flare, at the fenestration and at the distal part of the BECS, were calculated as the minimum BECS diameter divided by the maximum BECS diameter. Furthermore, the degree of stenosis was calculated as:

(1) $$Stenosis\;\left(\%\right)=\left(1-\frac{Dstenosis}{Dnormal}\right)\times 100\%$$

*D_stenosis_* was the mean of two orthogonal diameters at the most severe stenosed segment. *D_normal_* was the mean of two orthogonal diameters at the non-diseased segment of the same artery [12].

The third step in BECS analysis was to categorize the BECS-associated complications according to one or a combination of modes of failure as described in detail below (Endoleak (E1–E4), Obstruction (O1–O4), Fracture (F1–F4)). This was performed by a team consisting of one vascular surgeon (JV) and two researchers (RS and CR) during a consensus meeting.

### 2.4. BECS-Associated Endoleak Classification

**Figure 2:**
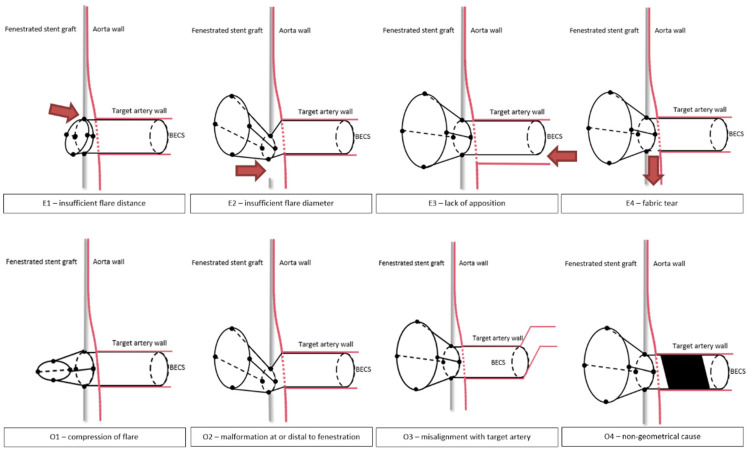
Schematic representation of modes of balloon-expandable covered stent (BECS) failure for endoleak and obstruction. The red arrows represent the blood flow in case of an endoleak.

A classification of potential modes of failure for BECS-associated endoleak is proposed based on an existing classification for endoleaks after FEVAR [13]. Oderich et al. reported three types of BECS-associated endoleaks: type 1c (between BECS and target artery), 3c (between BECS and FSG), and 3d (fabric tear). Based on the described geometric analysis of BECS, we modified the type 3c endoleak classification by making a distinction between too short flare-to-fenestration distance and lack of a circumferential seal between the BECS and the fenestration. From flare to target artery, the four modes of failure for a BECS-associated endoleak are at the proximal end of the flare (E1), at the level of the fenestration (E2), at the distal end of the BECS (E3), or at any location due to fabric tear or perforation (E4). The different modes of BECS failure are schematically shown in Figure 2 and described below:

E1. The first cause of endoleak is insufficient proportion of the BECS inside the FSG, resulting in an endoleak between the flared end of the BECS and FSG fabric. During the FEVAR procedure, this can occur due to inadequate deployment of the BECS. During follow-up, this can occur when the BECS migrates out of the FSG. The required reintervention exists of proximal BECS extension.
E2. The second cause of endoleak is a diameter mismatch between the flared end of the BECS and the fenestration, resulting in an endoleak. During the FEVAR procedure, this may occur during BECS deployment as a result of inadequate flaring or an undersized BECS compared to the dimensions of the fenestration, or dislocation of the BECS due to wire, catheter or delivery system maneuvers. During follow-up, this can occur as a result of BECS compression caused by FSG migration. The required reintervention exists of re-flaring the BECS and eventual proximal BECS extension.
E3. The third cause of endoleak is insufficient apposition between the distal part of the BECS and the target artery, resulting in blood flow into the aneurysm. This type of endoleak has previously been classified by Oderich et al. as a type 1c endoleak [13]. During the FEVAR procedure, this can occur due to insufficient BECS length or an undersized BECS diameter compared to the diameter of the target artery. During follow-up, this can occur due to BECS migration towards the aortic lumen. One of the underlying mechanisms for this is an increasing gap between the FSG and the aortic wall due to progression of disease or secondary to another endoleak that promotes aneurysm growth. The required reintervention exists of distal BECS extension.
E4. The fourth cause of endoleak is fabric tear. This type of endoleak has previously been classified by Oderich et al. as a type 3d endoleak [13]. During the FEVAR procedure, this can occur due to damage of the BECS fabric, for example by calcified arteries. During follow-up, this can be caused by a stent fracture resulting from BECS fatigue or FSG migration. The required reintervention exists of deployment of a new covered stent to seal the fabric tear or perforation.

### 2.5. BECS Obstruction Classification

A classification of potential modes of failure for BECS stenosis and occlusion is proposed partly based on different modes of BECS failure that have been described in literature. Two studies reported the flare ratio (stent diameter at the proximal end of the flare divided by the stent diameter at the level of the fenestration) [9,14]. Another study measured FSG misalignment which was associated with end-organ ischemia and/or death [15]. Furthermore, compression of stents in fenestrations have been described caused by FSG migration, consequently leading to a stent fracture [16]. We added a non-geometric mode of failure in case of in-stent thrombosis without stent frame stenosis. From flare to target artery, the four modes of failure for a BECS obstruction are at the proximal end of the flare (O1), at or distally to the level of the fenestration (O2), at the distal end of the BECS (O3), or at any location without a geometric BECS cause (O4). These modes of BECS failure are schematically shown in Figure 2:

O1. The first cause of stenosis or occlusion is compression of the flared part of the BECS. During the FEVAR procedure, this can occur due to insufficient or failed flaring, or dislocation of the BECS due to wire, catheter or delivery system maneuvers. During follow-up, this can be due to collapse of the flare, potentially caused by FSG migration [16]. The required reintervention should focus on re-flaring of the BECS, eventually with proximal extension.
O2. The second cause of stenosis or occlusion is BECS compression at the level of or distal to the fenestration, or blockage of the BECS inflow by the FSG fabric. During the FEVAR procedure, this can occur due to misalignment of the fenestration with the target artery. During follow-up, this can be caused by FSG migration, or the FSG fabric can obstruct the inflow of the target artery after complete FSG-BECS disconnection. The required reintervention exists of re-flaring the BECS and eventual proximal BECS extension with a new covered stent to strengthen the FSG-BECS connection.
O3. The third cause of stenosis or occlusion is misalignment of the distal end of the BECS with the target artery. This may cause turbulent flow patterns or obstruction [17]. During the FEVAR procedure, this can be due to insufficient BECS length and/or hostile target artery anatomy such as curvature and tortuosity [18]. Especially for the celiac trunk, this mode of failure can also be caused by median arcuate ligament compression [19]. The reintervention should focus on relining of the distal end of the BECS.
O4. The fourth cause of stenosis or occlusion is a non-geometric BECS related. Patient-related risk factors for BECS obstruction due to blood clotting and thrombus formation may be suspected when BECS geometry causes are ruled out. Low flow velocity results in low wall shear stress, which has been associated with thrombus formation [20]. In-stent thrombosis can occur at any time during follow-up.

### 2.6. BECS Fracture Classification

A BECS fracture was defined as at least one fractured strut that was diagnosed on CT or X-ray. BECS fractures may lead to clinical consequences and can occur in combination with endoleaks and/or obstructions. An existing stent fracture classification for stents in the superficial femoral artery has been applied to BECS used as a bridging stent in FEVAR procedures [21]:

F1. One single strut fracture;
F2. Multiple single strut fractures;
F3. Transverse linear BECS fracture without displacement;
F4. Transverse linear BECS fracture with displacement.

A reintervention for BECS fractures is necessary in case of clinically relevant complications, such as endoleak, thrombus formation, or hemodynamically significant stenosis.

### 2.7. Statistical Analysis

Data were analyzed using SPSS 27 statistical software (IBM Corp, Armonk, NY, USA). Normality of the data was assessed via visual inspection of Q-Q plots. Normally distributed variables were expressed as mean ± SD and skewed distributed variables were expressed as median (interquartile range). The difference in BECS-associated complications between the Zenith and Anaconda FSG was tested by a Fisher exact test. A probability (*p*) value <0.05 was considered as statistically significant.

## 3. Results

### 3.1. Patient Outcomes

This study included 279 FEVAR-patients (mean age, 72 ± 8 years; 86% male) and 683 fenestrations stented with an Advanta V12 BECS. Primary FEVAR was used in 258 patients (92%), and 21 patients (8%) were re-intervened with a fenestrated cuff after initial infrarenal EVAR. The median (interquartile range, IQR) surveillance duration with CTA or DUS was 21 (1−45) months.

A Zenith FSG (Cook Medical Inc, Bloomington, IN, USA) was used in 253 patients (91%), in which a total of 614 Advanta V12 BECSs were implanted. For these patients, 24 Advanta V12 BECS-associated complications (4%) were reported for 20 patients (8%). An Anaconda FSG (Terumo Aortic, Inchinnan, Scotland, UK) was used in 26 patients (9%), in which 69 Advanta V12 BECSs were implanted. For these patients, 10 Advanta V12 BECS-associated complications (14%) were reported for six patients (23%). There was a significantly (*p* < 0.001) higher rate of BECS-associated complications in patients treated with an Anaconda FSG compared to the group of Zenith FSG.

### 3.2. BECS Outcomes

**Figure A1:**
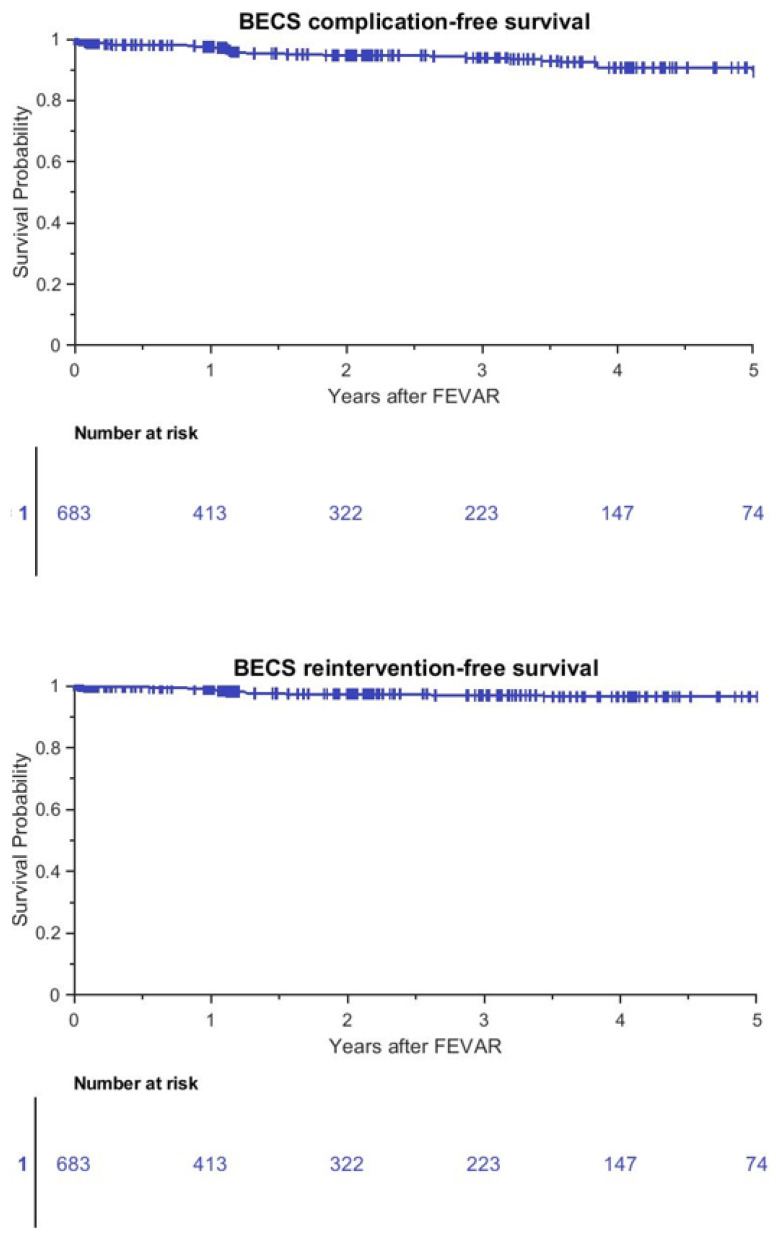
Kaplan–Meier curves for freedom from Advanta V12 balloon-expandable covered stent (BECS)-associated complication and reintervention. FEVAR, fenestrated endovascular aneurysm repair.

In 649 of 683 Advanta V12 BECSs (95%), there were no complications during follow-up. Freedom from Advanta V12 BECS-associated complications was 97% at 1 year and 94% at 3 years (Figure A1). Freedom from Advanta V12 BECS-associated reinterventions was 98% at 1 year and 97% at 3 years (Figure A1). The outcome of reinterventions is described in Table 1.

In 34 Advanta V12 BECSs (5%), there was a complication (Table 1). Two of these BECS-associated complications occurred during the FEVAR procedure and were classified as technical failure. One of these BECSs was open but with a small endoleak on the completion angiography which could not be solved. The other BECS occluded during the FEVAR procedure without possibilities to re-open. Post-FEVAR 3D geometric BECS analysis was not possible for five complicated BECSs due to missing or low-quality CTA scans.

The post-FEVAR CTA scans of the remaining 27 Advanta V12 BECSs had a median (IQR) slice thickness of 0.75 (0.75−0.75) mm. The median (IQR) time between the FEVAR procedure and the diagnosis of the BECS-associated complication was 14 (2−33) months. BECS geometry analysis based on a combination of CTA derived measurements and the FGA was performed to classify these 27 BECS-associated complications according to the modes of failure (E1–E4, O1–O4, F1–F4) as mentioned in the Section 2.

### 3.3. BECS-Associated Endoleak

**Figure 3:**
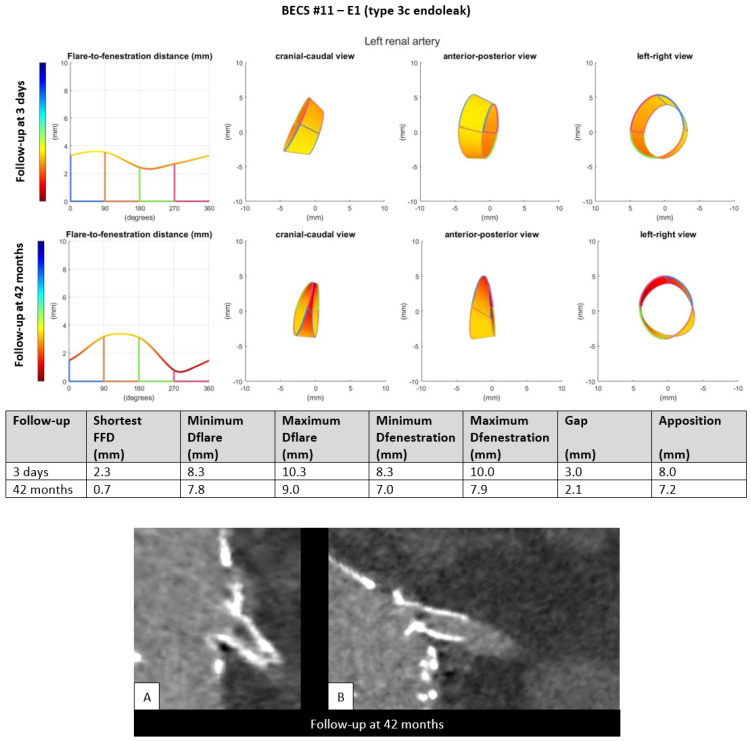
BECS #11 is an example of E1; endoleak due to insufficient flare distance. At the top, geometric analysis of 3-days and 42-months post-FEVAR CTA scans are shown. From left to right for both follow-up moments: circumferential FFD and cranial-caudal, anterior-posterior, and left-right view of the flare. At the bottom, (**A**) stretched vessel view of the aorta and (**B**) snake view of the left renal artery of the 42-months post-FEVAR CTA scan which showed the type 3c endoleak.

E1. Five BECSs had an endoleak due to insufficient length of the flare (FFD) or FSG-BECS disconnection and were categorized as E1 (Table 1, BECS #08, #09, #10, #11, and #12). BECS #11 is used as an example demonstrating this mode of failure (Figure 3). An endoleak of the BECS in the left renal artery was reported on the 42-months post-FEVAR CTA scan. Geometric analysis showed a shortest FFD of 2.3 mm on the 3-days post-FEVAR CTA scan and 0.7 mm on the 42-months post-FEVAR CTA scan, indicating BECS migration. The BECS was successfully relined with a proximal extension during reintervention.

**Figure 4:**
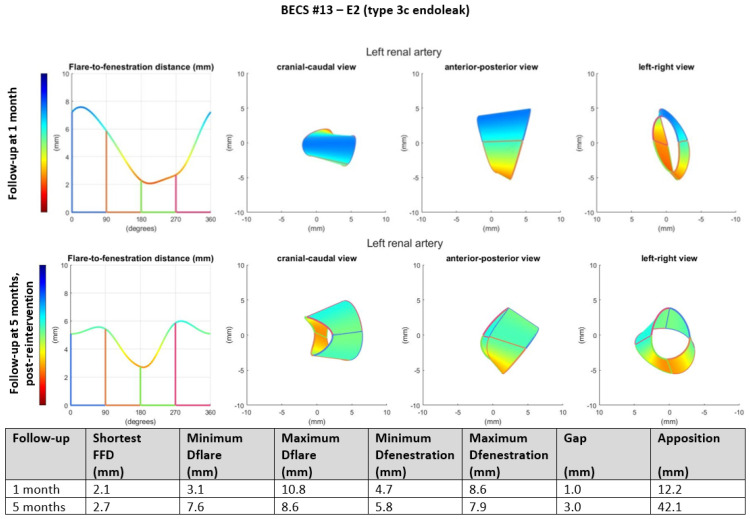
BECS #13 is an example of E2, endoleak due to insufficient flare. Geometric analysis was performed for the 1-month post-FEVAR CTA scan and post-reintervention CTA scan. Insufficient flaring of the proximal end of the BECS caused a type 3c endoleak on the 1-month post-FEVAR CTA scan. The geometric analysis shows an oval-shaped flare with a minimum diameter of 3.1 mm. The endoleak was treated successfully by renewed flaring and proximal extension. The geometric analysis of the post-reintervention CTA scan shows a circle-shaped flare with a minimum diameter of 7.6 mm.

E2. Two BECSs had an endoleak due to insufficient flare resulting in mismatch between the diameter of the BECS and the fenestration and were categorized as E2 and E2/O1 (Table 1, BECS #13 and #14, respectively). BECS #13 is used as an example demonstrating this mode of failure (Figure 4). An endoleak of the BECS in the left renal artery was reported on the 1-month post-FEVAR CTA scan. Geometric analysis of this post-FEVAR CTA scan showed minimum and maximum diameters of the flare of 3.1 mm and 10.8 mm, indicating an oval-shaped flare. Fenestration dimensions of the FSG were 6 × 8 mm and minimum and maximum diameters of the BECS at the fenestration were 4.7 mm and 8.6 mm, causing the endoleak. This BECS was successfully relined by renewed flaring and placement of a proximal extension.

**Figure 5:**
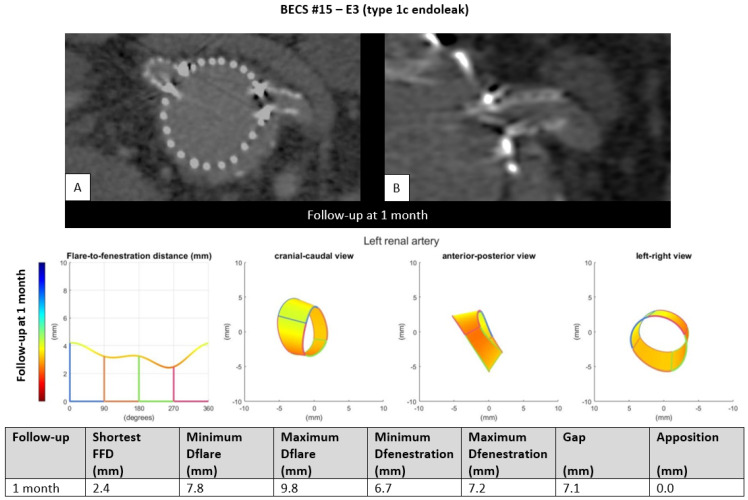
BECS #15 is an example of E3, endoleak due to insufficient circumferential apposition between the BECS and the target artery. The 1-month post-FEVAR CTA scan ((**A**); axial view of the aorta, (**B**); snake view of the left renal artery) reported a type 1c endoleak. Geometric analysis was performed of the 1-month post-FEVAR CTA scan and reported lack of apposition.

E3. One BECS had an endoleak due to loss of apposition between the BECS and the target artery and was categorized as E3 (Table 1, BECS #15). An endoleak of the BECS in the left renal artery was reported on the 1-month post-FEVAR CTA scan. Geometric analysis showed no circumferential apposition and a gap of 7.1 mm between FSG and aortic wall (Figure 5). This BECS was relined with an oversized balloon without resolving the endoleak. There was no further follow-up information available; the patient died from a stroke.

**Figure 6:**
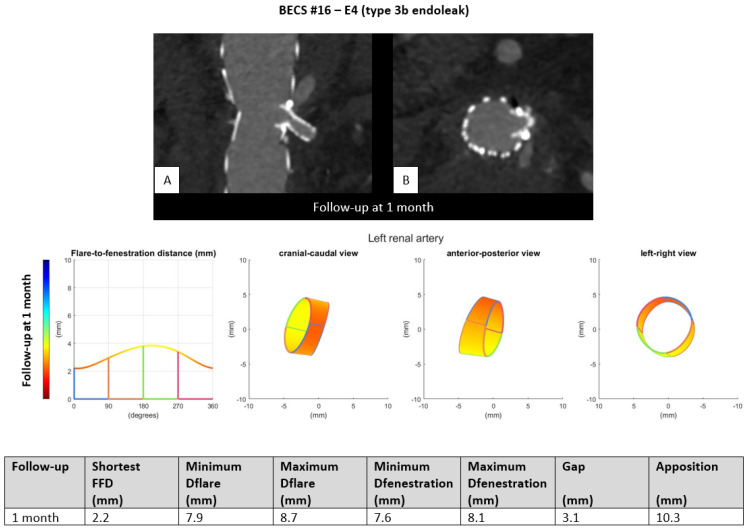
BECS #16 is an example of E4, endoleak due to fabric tear. The 1-month post-FEVAR CTA scan ((**A**); stretched vessel view of the aorta, (**B**); axial view of aorta) showed a flared BECS in the fenestrated stent graft, and the BECS is open. Geometric analysis of the 1-month post-FEVAR CTA scan showed no other cause that may explain the endoleak.

E4. One BECS had an endoleak that is most likely caused by fabric tear and was categorized as E4 (Table 1, BECS #16). An endoleak of the BECS in the left renal artery was reported on the 1-month post-FEVAR CTA scan (Figure 6). Geometric analysis showed a sufficiently positioned BECS with a D-ratio of 0.9 for both the flare and the fenestration, and 10.3 mm apposition. There was no reintervention performed because this patient suffered oncological disease with poor prognosis.

### 3.4. BECS Obstruction

**Figure 7:**
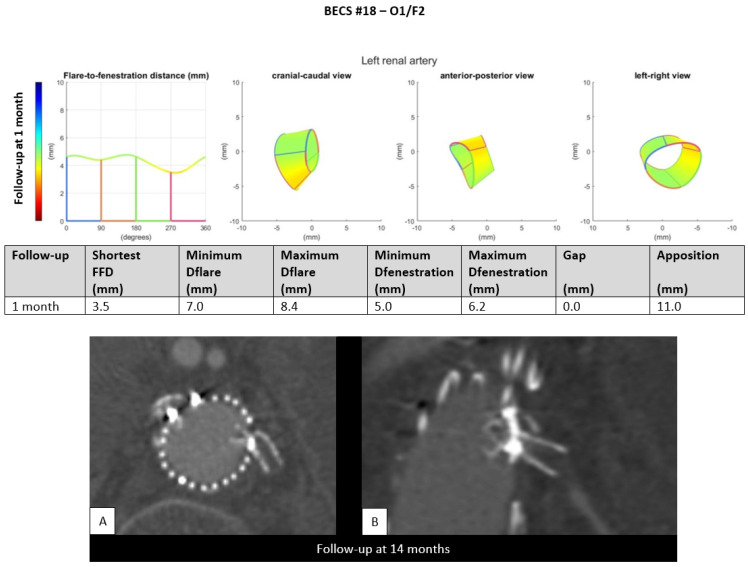
BECS #18 is an example of O1, combined with F2, obstruction due to flare compression and fracture of multiple struts. Geometric analysis of the 1-month post-FEVAR CTA scan shows a good flare with adequate diameters. The 14-months post-FEVAR CTA scan ((**A**); axial view of the aorta, (**B**); stretched vessel view of the aorta) showed a compressed and fractured BECS in the left renal artery.

O1. Two BECSs had compression of the flared end and were categorized as O1 and O1/F2 (Table 1, BECS #17 and #18, respectively). BECS #18 is used as an example demonstrating this mode of failure (Figure 7). This BECS in the left renal artery was occluded and fractured on the 14-month post-FEVAR CTA scan. A collapsed flare was seen on the snake view of the target artery, caused by FSG migration. There were no treatment options for this patient.

**Figure 8:**
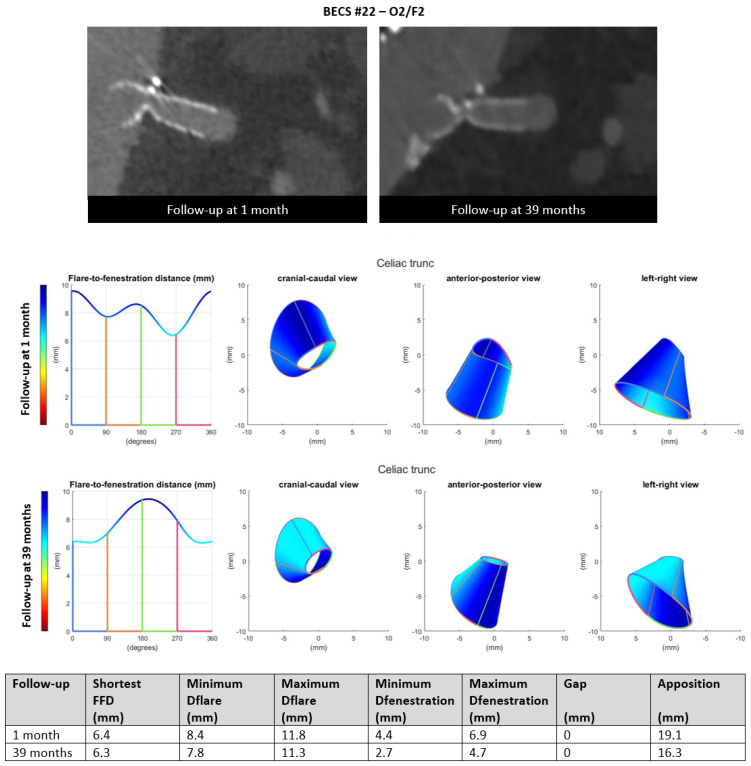
BECS #22 is an example of O2, combined with F2, obstruction due to compression at the fenestration and fracture of multiple struts. At the top, the snake view of 1-month and 39-months post FEVAR-CTA scans—at the bottom, geometric analysis of both follow-up moments, confirming the decreasing diameter at the height of the fenestration.

O2. Six BECSs had compression of the BECS at the level of the fenestration and were categorized; two as O2, three as O2/F2, and one as O2/F2/E1 (Table 1, BECS #19, #20, #21, #22, #23, and #24, respectively). BECS #22 is used as an example demonstrating this mode of failure (Figure 8). A 90% stenosis and type F2 fracture were reported for the BECS in the celiac trunk on the 39-months post-FEVAR CTA scan. Geometric analysis showed minimum and maximum diameter of fenestration of 4.4 by 6.9 on the 1-month post-FEVAR CTA scan, which decreased to 2.7 by 4.7 mm on the 39-months post-FEVAR CTA scan. BECS compression at the level of the fenestration was caused by FSG migration. A successful reintervention with Endoanchors was performed to prevent further FSG migration. There was no reintervention of the BECS complication performed due to technical difficulties.

**Figure 9:**
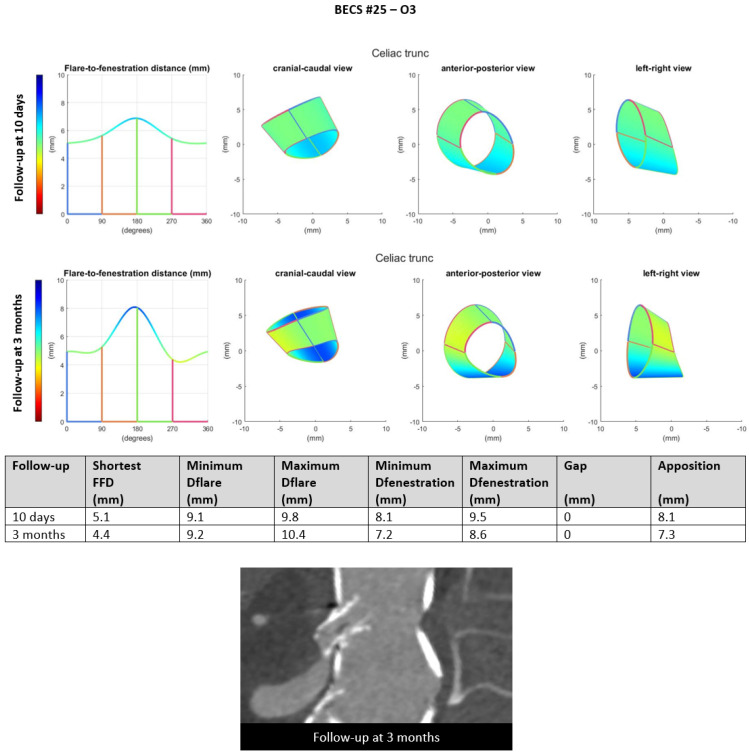
BECS #25 is an example of O3, obstruction due to misalignment or constriction of the distal end of the BECS with the target artery. At the top, geometric analysis of the 10-days and 3-months post-FEVAR CTA scans showed a good flare without major geometrical changes. At the bottom, the snake view of the 3-month post-FEVAR CTA scan showed 80% stenosis distal to the BECS due to median arcuate ligament compression.

O3. One BECS was categorized as O3 (Table 1, BECS #25). This BECS in the celiac trunk had an 80% stenosis distal of the BECS on the 3-months post-FEVAR CTA scan (Figure 9). This was caused by median arcuate ligament compression. Geometric analysis showed subtle changes in BECS geometry between the 10-days and 3-months post-FEVAR CTA scan. There was no reintervention indicated by the physicians as the patient was symptom free and the SMA was patent with adequate collaterals to the distal celiac trunk branches.

**Figure 10:**
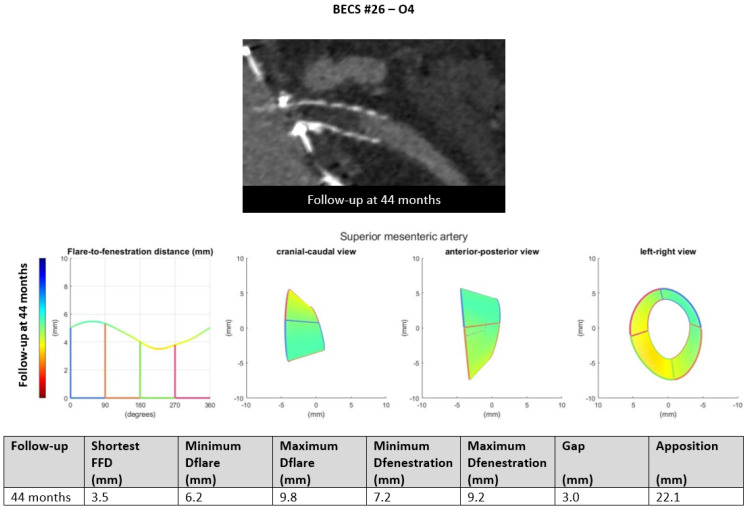
BECS #26 is an example of O4, obstruction without a geometrical cause. The 44-month post-FEVAR CTA scan reported an occluded BECS. Geometric analysis showed no abnormalities of the BECS geometry that may have caused the occlusion.

O4. Six BECSs had no geometric cause and were categorized as O4 (Table 1, BECS #26, #27, #28, #29, #30, and #31). BECS #26 is used as an example demonstrating this mode of failure (Figure 10). This BECS in the SMA was occluded on the 44-month post-FEVAR CTA scan. Geometric analysis showed no abnormalities of the BECS stent frame itself. The occlusion was caused by thrombosis due to confirmed cryoglobulinemia type 2. No reintervention was performed as the patient refused treatment and subsequently died as a result of multi-organ failure.

### 3.5. BECS Fracture

Three BECS were fractured without clinical consequences (Table 1, BECS #32, #33, and #34). BECS #32 in the LRA showed a type 1 fracture on the 64-month abdominal X-ray. BECS #33 in the SMA showed a type 2 fracture on the 15-month post-FEVAR CTA scan. BECS #34 in the RRA showed a type 4 fracture on the 35-month abdominal X-ray. The cause of this type 4 fracture was migration of the Anaconda FSG possibly due to a fractured proximal stent. All three BECSs were open without an endoleak and/or obstruction on CTA follow-up.

## 4. Discussion

In this paper, the classification system for post-FEVAR BECS-associated complications was refined. All Advanta V12 BECS from a bi-center dataset of FEVAR-patients were assessed for BECS-associated endoleak, stenosis, obstruction, and fracture. Freedom from Advanta V12 BECS-associated complications was 97% at 1 year and 94% at 3 years, and freedom from Advanta V12 BECS-associated reinterventions was 98% at 1 year and 97% at 3 years. Of the 5% BECSs with a complication for which a CTA was available, all could be classified according to the classification system.

It can be difficult to find the origin of the endoleak on a standard CTA scan. 3D geometric analysis of the BECS (i.e., short FFD, insufficient diameters of the flare or at the fenestration, abnormal D-ratios, lack of distal apposition) helps to better differentiate between these causes, which is needed to determine the correct reintervention strategy. As an example, BECS #15 was reintervened with an oversized balloon to obtain an optimal distal seal, but this was unsuccessful. With the current geometric analysis, it seems that the BECS was too short (22 mm). A distal extension would have been a more appropriate solution.

BECS obstruction without aberrant BECS geometry was observed for 6 out of 16 obstructed BECSs (38%). Tran et al. also found that a post-FEVAR reduction in aortic wall shear stress and increased particle residence time correlated with the development of intraluminal graft thrombus and renal stent occlusion in patients treated with a large diameter (>34 mm) FSG [20]. Another possible explanation for BECS obstruction without evidence of BECS compression is antiplatelet drug resistance.

Assessing the flare in 3D is challenging on standard intraoperative angiography. Cone-beam CTA at the end of the FEVAR procedure may allow for better quantifying the flare geometry intraoperatively and may therefore enable intraoperative revision if needed. However, cone-beam CT increases the procedural costs, use of contrast and adds to radiation exposure; thus, its added value should be proven first [22,23]. Intravascular ultrasound (IVUS) is another potential technique to detect branch instability that is missed by angiography [24]. However, these promising developments will not resolve all postoperative bridging stent complications that occur in 20% of FEVAR-patients and in 8% of stents at median (IQR) follow-up of 3.7 years (1.7−5.3) [6]. Correct classification of the endoleak or obstruction on post-FEVAR CTA imaging remains essential to determine the exact cause of the failure and hence choose the appropriate reintervention.

Geometric changes of the BECS may prelude complications before an actual endoleak or occlusion occurs. There are, however, no definitions in the guidelines of the ideal flare-to-fenestration distance and sufficient amount of flaring of the BECS to secure a good connection, neither is there a definition of sufficient distal apposition with the target artery. Further studies should establish cut-off values for sufficient flare-to-fenestration distance, sufficient flare-to-fenestration diameter ratio, and sufficient apposition with the target artery. We suggest that geometrical aspects of the BECS should be reported in a standard way for studies assessing FEVAR complications.

In this study, two different types of FSGs have been used; Cook Zenith and Anaconda. Advanta V12 BECS-associated complications were significantly lower in patients treated with a Zenith FSG compared to an Anaconda FSG. Literature reported target vessel patency at three years of 92.7% ± 1.4% for fenestrations of the Anaconda FSG and 98.1% ± 0.6% for fenestrations of the Cook Zenith FSG, but no randomized trial has been performed so far [2,25]. A possible explanation for differences in outcomes might be the difference in FSG design. The Anaconda FSG consists of an unsupported body distal of the two parallel proximal rings which may increase its conformability to the aortic shape compared to the Zenith FSG. The unsupported Anaconda FSG body may, however, also influence (and increase) the dynamic forces on the fenestrations and BECS especially if the FSG is not fully opposed to the aortic wall. Further research should investigate the influence of geometrical changes of the fenestrations and BECS geometry in different types of fenestrated endografts and various aortic anatomies.

One limitation of this study is that CTA-based geometric analysis was only performed for BECS with a complicated follow-up. BECS without complicated follow-up were assessed only from data in the electronic patient files. Thence, cut-off values for geometrical BECS parameters could not be determined with the data in this study. A second limitation is the retrospective design of this study. Some patients were lost to follow-up as a result of out-of-region surveillance; therefore, not all BECS-associated complications may have been included.

A limitation of the 3D geometric analysis is that BECS parameters were calculated from manual measurements in the 3mensio vascular workstation and are therefore subjective to measurement variability. A previous validation study showed that FFD and BECS diameters could be determined with 2-mm and 1-mm measurement variability, respectively [10]. A change in the angle between the target artery and the BECS has been associated with target artery occlusion, but this variable has not been measured in our study, which can be considered a limitation [16]. In addition, fabric tear (E4) and non-geometric causes for obstruction (O4) cannot be verified with the geometric analysis of the BECS on CTA. It may therefore be suspected when other causes are ruled out. As a last limitation, the FGA software is not CE/FDA marked yet. Therefore, clinical implementation of these calculations is not yet possible. As an alternative, distances and diameters can be measured over the centerline in a vascular workstation, but the accuracy of these measurements and relationship with the parameters calculated by the FGA software have not been verified.

## 5. Conclusions

A classification system consisting of twelve different BECS-associated complications has been proposed that aids in determining the correct reintervention strategy. Three of four causes for both endoleak and obstruction can be geometrically assessed with CTA-derived measurements and flare geometry analysis. Fifty nine percent of the BECS had an uneventful follow-up, and all BECS with a complication could be categorized based on available CTA scans.

## Figures and Tables

**Table 1:** Overview of studied variables per Advanta V12 balloon-expandable covered stent (BECS) with complicated follow-up.

BECS ### Figure Number	FSG Device	Target Artery	Size of Fenestration (Width × Height, mm)	Size of BECS (Diameter × Length, mm)	Time between FEVAR and First Post-FEVAR CTA (Months)	Time between FEVAR and Complication (Months)	Complication or Combined Complications	Mode(s) of Failure	Cause	Reintervention (Yes/No; Explanation)
BECS #01	Zenith	LRA	6 × 8	7 × 22	*N.A.*	0	Endoleak	*Technical failure*	Unknown	No; patient refused further treatment
BECS #02	Zenith	RRA	6 × 8	7 × 22	*N.A.*	0	Occlusion	*Technical failure*	Unknown	No
BECS #03	Zenith	LRA	6 × 8	7 × 22	*Only DUS*	10	Occlusion	*Unknown (no CTA scan)*	Unknown	Yes; recanalization but unsuccessful. Afunctional kidney
BECS #04 ^a^	Zenith	RRA	6 × 8	6 × 22	*Only DUS*	13	Stenosis	*Unknown (no CTA scan)*	Unknown	Yes; extra stent and PTA, success
BECS #05 ^a^	Zenith	LRA	6 × 8	6 × 22	*Only DUS*	13	Occlusion	*Unknown (no CTA scan)*	Unknown	Yes; recanalization and extra stent, success
BECS #06	Zenith	LRA	6 × 8	6 × 22	2	14	Stenosis	*CTA scan not suitable for geometric analysis*	Unknown	No; no renal insufficiency
BECS #07	Zenith	RRA	6 × 8	5 × 22	<1	19	Fracture and stenosis	*CTA scan not suitable for geometric analysis*	Unknown	Yes; extra stent, success
BECS #08	Zenith	SMA	8 × 8	8 × 38	1	32	Endoleak	E1	BECS migration	Yes; proximal extension, success
BECS #09 ^b^	Zenith	RRA	6 × 8	6 × 22	<1	7	Endoleak	E1	BECS migration, due to FSG migration due to new dissection of suprarenal aorta	Yes; proximal extension, success
BECS #10	Zenith	SMA	8 × 8	8 × 38	1	14	Endoleak	E1	BECS migration	Yes; proximal extension, success
BECS #11 Figure 3	Zenith	LRA	6 × 8	6 × 22	<1	42	Endoleak	E1	BECS migration	Yes; proximal extension, success
BECS #12	Zenith	LRA	6 × 8	5 × 22	3	3	Endoleak	E1	BECS malposition (during FEVAR procedure)	No; oncological disease with short life expectancy
BECS #13 Figure 4	Zenith	LRA	6 × 8	5 × 22	1	1	Endoleak	E2	Technical failure at flared end	Yes; renewed flaring and extra stent, success
BECS #14	Zenith	LRA	6 × 8	6 × 22	<1	<1	Endoleak and 70% stenosis	E2/O1	Technical failure at flared end	Yes; renewed flaring and extra stent, success
BECS #15 Figure 5	Zenith	LRA	6 × 8	6 × 22	1	1	Endoleak	E3	Too short BECS (sizing problem)	Yes; re-ballooned but unsuccessful. Patient died due to other reason
BECS #16 Figure 6	Zenith	LRA	6 × 8	7 × 22	1	1	Endoleak	E4	Probably fabric tear	No; oncological disease with short life expectancy
BECS #17	Zenith	LRA	6 × 8	7 × 32	1	1	50% stenosis	O1	Technical failure at flared end	No; without clinical consequences, DUS follow-up
BECS #18 Figure 7	Zenith	LRA	6 × 8	6 × 22	1	14	Occlusion and fracture	O1/F2	Flare compression due to migration/rotation of FSG	No; no reintervention options
BECS #19 ^c^	Zenith	RRA	6 × 8	7 × 38	1	3	60% stenosis	O2	Compression at fenestration	Yes; re-flaring, success
BECS #20	Anaconda	Right ARA	6 × 6	5 × 22	2	2	Occlusion	O2	FSG-BECS disconnection	No; there were no treatment options and no clinical consequences (ARA)
BECS #21 ^d^	Anaconda	LRA	7 × 7	6 × 22	<1	14	70% stenosis and fracture	O2/F2	FSG migration	Yes; re-PTA and additional stent, success
BECS #22 Figure 8	Anaconda	TRU	9 × 9	8 × 32	1	39	90% stenosis and fracture	O2/F2	FSG migration	No; asymptomatic stenosis and no reintervention options
BECS #23 ^d^	Anaconda	TRU	7 × 7	6 × 22	<1	44	Occlusion and fracture	O2/F2	FSG migration, leading to upward facing BECS with compression and fracture	No; no clinical consequences and no reintervention options
BECS #24 ^b^	Zenith	SMA	8 × 8	8 × 38	<1	7	90% stenosis, fracture, and endoleak	O2/F2/E1	FSG migration due to new dissection of suprarenal aorta	Yes; extra stent, success
BECS #25 ^e^ Figure 9	Zenith	TRU	8 × 8	6 × 22	<1	3	80% stenosis distal of the BECS	O3	Too short BECS, ending in tortuous TRU	No; asymptomatic stenosis
BECS #26 Figure 10	Zenith	SMA	8 × 8	9 × 38	<1	44	Occlusion	O4	Thrombosis, cryoglobulin type 2	No; patient refused treatment and died due to MOF
BECS #27	Anaconda	SMA	8 × 8	7 × 38	1	22	50% lumen reduction due to thrombus in BECS	O4	Thrombosis ECI	No; after 6 months DUS follow-up, with stationary 50% in-BECS lumen reduction due to thrombus
BECS #28 ^f^	Anaconda	TRU	7 × 7	7 × 32	1	11	Occlusion	O4	Thrombosis ECI	No; patient not suitable for reintervention, died due to MOF
BECS #29 ^f^	Anaconda	RRA	8 × 8	6 × 32	1	47	Occlusion	O4	Progression of FSG thrombosis ECI	No; patient not suitable for reintervention, died due to MOF
BECS #30 ^f^	Anaconda	LRA	9 × 9	6 × 32	1	47	Occlusion	O4	Progression of FSG thrombosis ECI	No; patient not suitable for reintervention, died due to MOF
BECS #31 ^f^	Anaconda	SMA	10 × 10	9 × 32	1	47	Occlusion	O4	Progression of FSG thrombosis ECI	No; patient not suitable for reintervention, died due to MOF
BECS #32 ^c^	Zenith	LRA	6 × 8	7 × 38	1	64	Fracture	F1	ECI	No; open BECS on CTA
BECS #33 ^e^	Zenith	SMA	8 × 8	8 × 32	<1	15	Fracture	F2	ECI	No; open BECS on CTA
BECS #34	Anaconda	RRA	7 × 7	6 × 22	1	35	Fracture	F4	FSG migration	No; open BECS on CTA and DUS, no good reintervention options

^a^ BECS #04 and #05 are in the same patient. ^b^ BECS #09 and #24 are in the same patient. ^c^ BECS #19 and #32 are in the same patient. ^d^ BECS #21 and #23 are in the same patient. ^e^ BECS #25 and #33 are in the same patient. ^f^ BECS #28, #29, #30, and #31 are in the same patient. Abbreviations: N.A., not applicable; ECI, e causa ignota; FEVAR, fenestrated endovascular aneurysm repair; FSG, fenestrated stent graft; CTA, computed tomography angiography; LRA, left renal artery; MOF, multi-organ failure; PTA, percutaneous transluminal angioplasty; ARA, accessory renal artery, RRA, right renal artery; SMA, superior mesenteric artery; TRU, celiac trunk; DUS, duplex ultrasonography.

## Data Availability

Not applicable.
